# Identification of serum protein biomarkers for clear cell renal cell carcinoma using patient-derived xenografts

**DOI:** 10.1172/JCI193567

**Published:** 2025-11-04

**Authors:** Dalin Zhang, Chun-Lung Chiu, Fernando Jose Garcia Marques, Abel Bermudez, Christian R. Hoerner, Nicholas Hadi, Elise Wang, Thomas J. Metzner, Ludimila Trabanino, John T. Leppert, Hongjuan Zhao, Robert Tibshirani, Alice C. Fan, Sharon J. Pitteri, James D. Brooks

**Affiliations:** 1 Department of Urology, Stanford University School of Medicine, Stanford, California, USA.; 2 Department of Veterinary Medicine, College of Veterinary Medicine, National Pingtung, University of Science and Technology, Pingtung, Taiwan.; 3 Department of Radiology,; 4 Canary Center,; 5 Department of Medicine, Division of Oncology, and; 6 Department of Biomedical Data Sciences, Stanford University School of Medicine, Stanford, California, USA; 7 Department of Statistics, School of Humanities and Sciences, Stanford, California, USA.

**Keywords:** Nephrology, Oncology, Biomarkers, Cancer, Proteomics

## Abstract

We have identified a serum biomarker panel for clear cell renal cell carcinoma that suitable for clinical applications such as diagnosis and monitoring treatment response.

**To the Editor:** The diagnosis and management of clear cell renal cell carcinoma (ccRCC) rely almost entirely on medical imaging, particularly computerized tomography, MRI, and ultrasound. In several instances, medical imaging is inadequate or misleading, such as in assessing whether lesions less than 3 cm are benign or malignant, and in monitoring responses to immunotherapy, where tumors responding to therapy can grow due to immune infiltrates, so-called pseudoprogression. These challenges could be mitigated by the development of serum biomarkers; however, discovery and validation of serum DNA, RNA, and protein biomarkers have failed due to poor sensitivity, lack of reproducibility, and failed validation (1). Challenges to serum biomarker discovery include the presence of a high abundance of serum proteins and nucleotides that overwhelm discovery efforts by obscuring the cancer-derived secreted proteins, uncertainties about which of the many proteins that differ between cancer and normal kidney tissues are secreted into the serum, and the relatively low levels of the analytes released from the cancer.

To overcome these challenges, we used an approach employed by Sinha et al. (2), where sera from patient-derived xenograft (PDX) mouse models was analyzed to identify “human-specific” proteins (HSPs) that, by definition, are secreted or shed by the human tumor grafts and can be distinguished from mouse proteins based on their sequence differences (Figure 1A). We used 8 ccRCC PDX lines representing a range of RCC sites (primary tumor and metastases), histologic features (tumor grade, sarcomatoid/rhabdoid features), and VHL mutation status (Supplemental Figure 1A). When the xenograft tumor volume reached approximately 1.5 cm^3^, determined by MRI (3), sera and tumor tissues we harvested from 3 mice per PDX line, and proteomic profiling was performed as described previously (4). We identified 785 proteins that were significantly higher in ccRCC PDX tissue compared with 32 normal human kidney tissues (Figure 1B). We then identified 123 HSPs present in the sera of at least 3 PDX that were not detectable in control mouse sera (Figure 1C). When these 2 datasets were overlapped with the Secretome database, 7 common proteins with diverse cellular locations and functions were identified for further validation (Figure 1D and Supplemental Figure 1B). KEGG pathway enrichment analysis demonstrated that these proteins are involved in RCC-relevant biological processes such as glycolysis (Supplemental Figure 1C).

All 7 candidates showed higher expression in 110 ccRCC tissues compared with 84 adjacent normal tissues from the CPTAC ccRCC Discovery and Confirmatory Study (PDC000200 version 2) (Supplemental Figure 2A). Protein levels of LGALS1 positively correlated with increasing ccRCC stage in this cohort (Supplemental Figure 2B). All 7 markers also showed high expression in sera from PDX-bearing mice and were low or undetectable in sera from non-PDX–bearing mice by ELISA (Figure S3A). In addition, serum levels of 5 candidates were associated with tumor weight in 6 PDX lines (Supplemental Figure 3B).

Next, we performed ELISAs on sera from 49 patients with histologically confirmed ccRCC and 34 patients without RCC (including 8 patients diagnosed with oncocytoma and 2 with angiomyolipoma, both benign kidney tumor types, while the remaining samples were from kidney donor candidates free of renal neoplasms on imaging) (Supplemental Table 1). Levels of MIF, CTSD, GPI, PPIB, and CUTA were significantly higher in sera of ccRCC patients compared with non-RCC sera (Figure 1E). LAMC1 and LGALS1 also showed elevated levels in ccRCC patient sera compared with non-RCC sera, albeit differences were not statistically significant (Figure 1E). Moreover, the serum levels of 5 candidates were associated with ccRCC stage (Supplemental Figure 3C), and serum levels of 3 candidates were associated with tumor size (Supplemental Figure 3D).

To assess the performance of these candidate markers, we built a sparse linear regression model using Least Absolute Shrinkage and Selection Operator (LASSO) (5) that was trained on 65% of the data (32 ccRCC and 22 non-RCC) and tested on 35% of the data (17 ccRCC and 12 non-RCC); samples were assigned to training and test groups by stratified random sampling to ensure the class proportions were identical in both groups. A risk score using serum levels of 6 candidate markers (MIF, LGALS1, PPIB, CTSD, CUTA, GPI) distinguished ccRCC from non-RCC with an AUC of 0.86 (90% confidence interval: 0.77–0.95) and 0.88 (90% confidence interval: 0.78–0.98) with 5-fold cross validation in the training and test datasets, respectively (Figure 1F). These results demonstrate that our predictive model can accurately distinguish ccRCC from non-RCC in our cohort.

We have identified a parsimonious serum biomarker panel for ccRCC with excellent performance characteristics, and that is potentially suitable for clinical applications. Our predictive model could help ccRCC diagnosis when renal mass is detected incidentally. This could spare patients with benign renal masses from unnecessary surgery, distinguish true from pseudoprogression in patients treated with ICIs, and could potentially be useful in monitoring response to other therapies. Additional testing in discrete patient cohorts will be necessary to determine utility in these specific clinical applications.

## Funding support

This work is the result of NIH funding, in whole or in part, and is subject to the NIH Public Access Policy. Through acceptance of this federal funding, the NIH has been given a right to make the work publicly available in PubMed Central.

- NIH NCI grants 1R21CA256271 and 5R21CA276896.
- Department of Defense grant W81XWH2210651.

## Supplementary Material

Supplemental data

Supporting data values

## Figures and Tables

**Figure 1:**
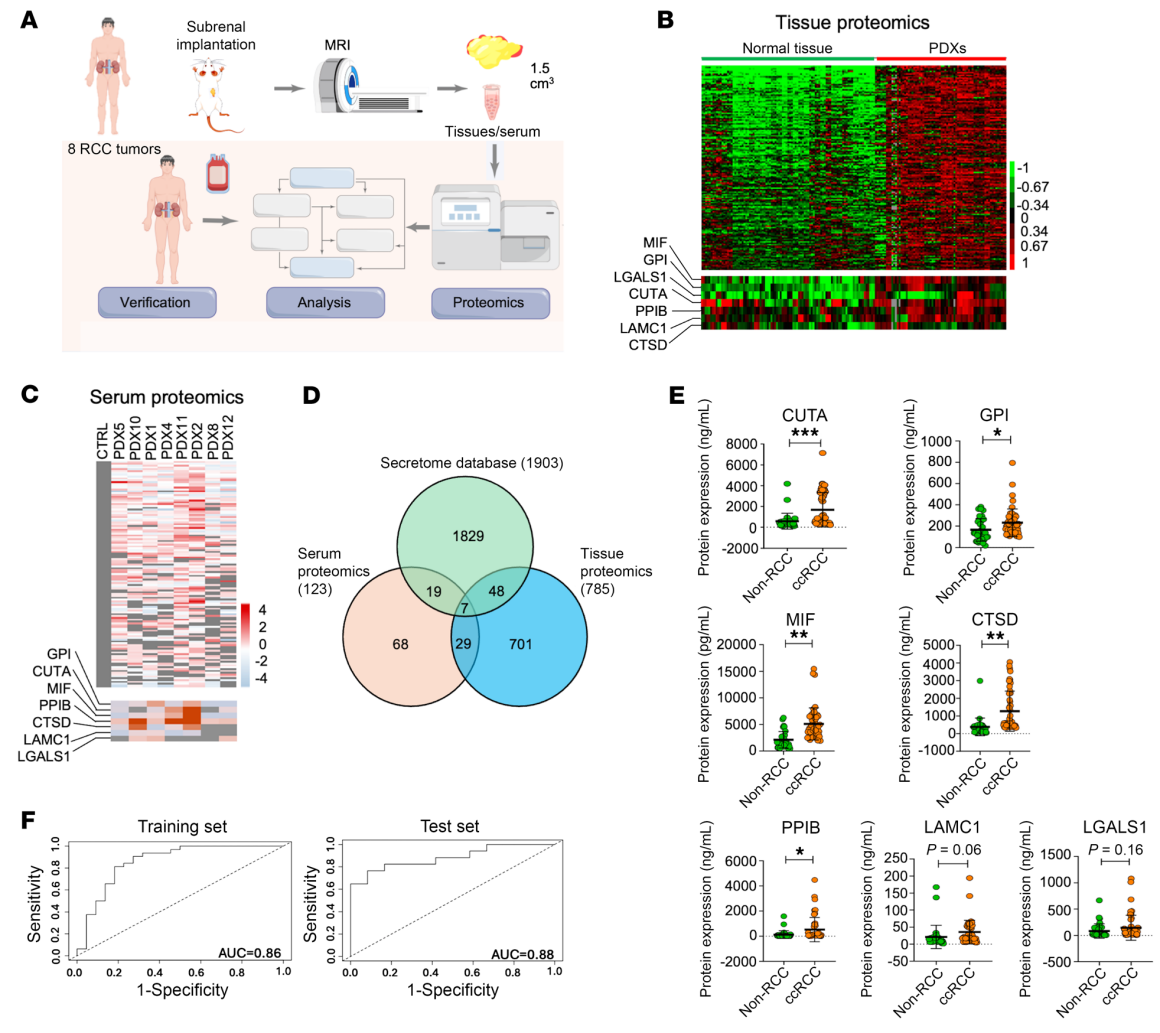
Discovery and validation of serum protein biomarkers in ccRCC. (**A**) The experimental workflow of our study. (**B**) Proteins with significantly higher levels in ccRCC PDX tissues compared with normal human kidney tissues. (**C**) Proteins detected in tumor-bearing mouse sera but not control sera. (**D**) Seven common proteins from **B** and **C** that are found in the Secretome database. (**E**) Validation of 7 candidate markers in sera from 34 non-ccRCC donors and 49 patients with ccRCC by ELISA. After Bonferroni adjustment for multiple comparisons, CUTA, MIF, and CTSD remain significant at *P =* 0.05. (**F**) AUCs of a linear-regression model based on levels of 6 candidate serum markers in distinguishing ccRCC from non-RCC. *P < 0.05; **P < 0.01; ***P < 0.001.

## Version 1 11/04/2025

In-Press Preview

## Version 2 01/02/2026

Electronic publication
