# Supplementary material for: Identification of serum protein biomarkers for clear cell renal cell carcinoma using patient-derived xenografts
Source: J Clin Invest. 2025 Nov 4;136(1):e193567. doi: 10.1172/JCI193567 (PMC12721905; doi:10.1172/JCI193567)
Supplement: Supplemental data [file jci-136-193567-s130.pdf]

Supplemental material

A

ID	Patient Sex	Patient Age	Patient Race	Source Tumor Site	Primary Tumor or Metastasis	Source of Tumor Tissue	Source RCC Tumor Histologic Subtype	Patient Compound RCC Stage	Patient TNM RCC Stage	Sarcomatoid Differentiation in Source Tumor	Rhabdoid Differentiation in Source Tumor	Source Tumor Grade	VHL Status	Treatment History
PDX1	M	74	Unknown	Kidney	Primary Tumor	Nephrectomy	Mixed (Clear Cell-like, Papillary-like, and Chromophobe-like)	4	pT2a pNx cN0 cM1	Present	Unknown	4 (Fuhrman)	MUT	Treatment-Naïve
PDX2	F	73	White	Kidney	Primary Tumor	Nephrectomy	Clear Cell	4	pT3a pNx cN0 pM1	Present	Unknown	4 (Fuhrman)	MUT	Treatment-Naïve
PDX3*	M	44	White	Sigmoid Colon	Metastasis	Metastasectomy	Clear Cell	4	cT0 cN1 pM1	Unknown	Unknown	Unknown	MUT	Treatment-Naïve
PDX4	M	71	White	Kidney	Primary Tumor	Nephrectomy	Clear Cell	4	pT3a pN0 cN0 pM1	Not Identified	Unknown	3 (Fuhrman)	MUT	Treatment-Naïve
PDX5	F	58	White	Liver	Metastasis	Autopsy	Clear Cell	4	cT0 cN0 pM1	Unknown	Unknown	Unknown	WT	Treatment-Naïve
PDX6	F	73	Unknown	Kidney	Primary Tumor	Nephrectomy	Clear Cell	4	pT3b pNx cN0 pM1	Present	Unknown	4 (WHO/ISUP)	MUT	Treatment-Naïve
PDX8*	M	49	White	Kidney	Primary Tumor	Nephrectomy	Clear Cell	Unknown	Unknown	Unknown	Unknown	Unknown	MUT	Treatment-Naïve
PDX10	M	59	Unknown	Kidney	Primary Tumor	Nephrectomy	Clear Cell	3	pT3a cN1 cM0	Not Identified	Not Identified	4 (WHO/ISUP)	MUT	Treatment-Naïve
PDX11	M	62	Asian	Lung	Metastasis	Metastasectomy	Clear Cell	4	cT0 cN1 pM1	Not Identified	Present	4 (WHO/ISUP)	MUT	Cabozantinib; Ipilimumab plus Nivolumab
PDX12	M	62	White	Retroperitoneal Lymph Node	Metastasis	Biopsy	Clear Cell	4	cT3a pN1 cM1	Not Identified	Not Identified	3 (WHO/ISUP)	MUT	Treatment-Naïve
PDX13*	M	60	Asian	Lung	Metastasis	Biopsy	Clear Cell	4	cT0 cN0 pM1	Unknown	Unknown	Unknown	MUT	Axitinib plus Pembrolizumab

*not used in proteomic analysis, only used in validation by ELISA

B

Gene	Location	Functions
**PPIB**	Cytosol/Endoplasmic reticulum/ extracellular etc.	Associated with the secretory pathway and released in biological fluids. Bind to cells derived from T- and B-lymphocytes and may regulate cyclosporine A mediated immunosuppression.
**LGALS1**	Extracellular/ cytosol/ endoplasmic reticulum etc.	The galectins are beta-galactoside-binding proteins implicated in modulating cell-cell and cell-matrix interactions. It may act as an autocrine negative growth factor that regulates cell proliferation.
**CUTA**	Extracellular/ mitochondrion etc.	Enables enzyme binding activity. Involved in protein localization. Located in membrane.
**CTSD**	Lysosome/ extracellular etc.	The encoded preproprotein is proteolytically processed to generate multiple protein products. This enzyme exhibits pepsin-like activity and plays a role in protein turnover and in the proteolytic activation of hormones and growth factors.
**MIF**	Cytosol/ extracellular etc.	This gene encodes lymphokine involved in cell-mediated immunity, immunoregulation, and inflammation. It can regulate macrophage function in host defense through the suppression of anti-inflammatory effects of glucocorticoids.
**LAMC1**	Endoplasmic reticulum/ extracellular etc.	Laminins, a family of extracellular matrix glycoproteins. They have been implicated cell adhesion, differentiation, migration, signaling, neurite outgrowth and metastasis.
**GPI**	Cytosol/ plasma membrane etc.	This gene encodes a member of the glucose phosphate isomerase protein family. Extracellularly, the protein functions as a neurotrophic factor that promotes survival of skeletal motor neurons and sensory neurons, and as a lymphokine that induces immunoglobulin secretion.

C

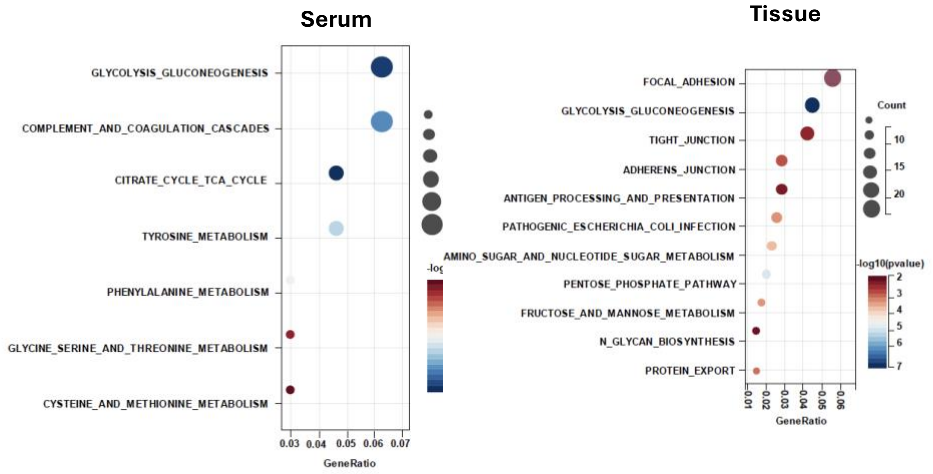

Figure S1 (A) ccRCC PDX lines used in this study and corresponding clinical information of the source tumors; (B) Pathway enrichment analysis using proteins detected in tumor-bearing mouse sera but not control sera; (C) KEGG pathway enrichment analysis of proteins with significantly higher levels in ccRCC PDX tissues compared to normal human kidney tissues; (D) Cellular location and function of each of the 7 candidate markers.

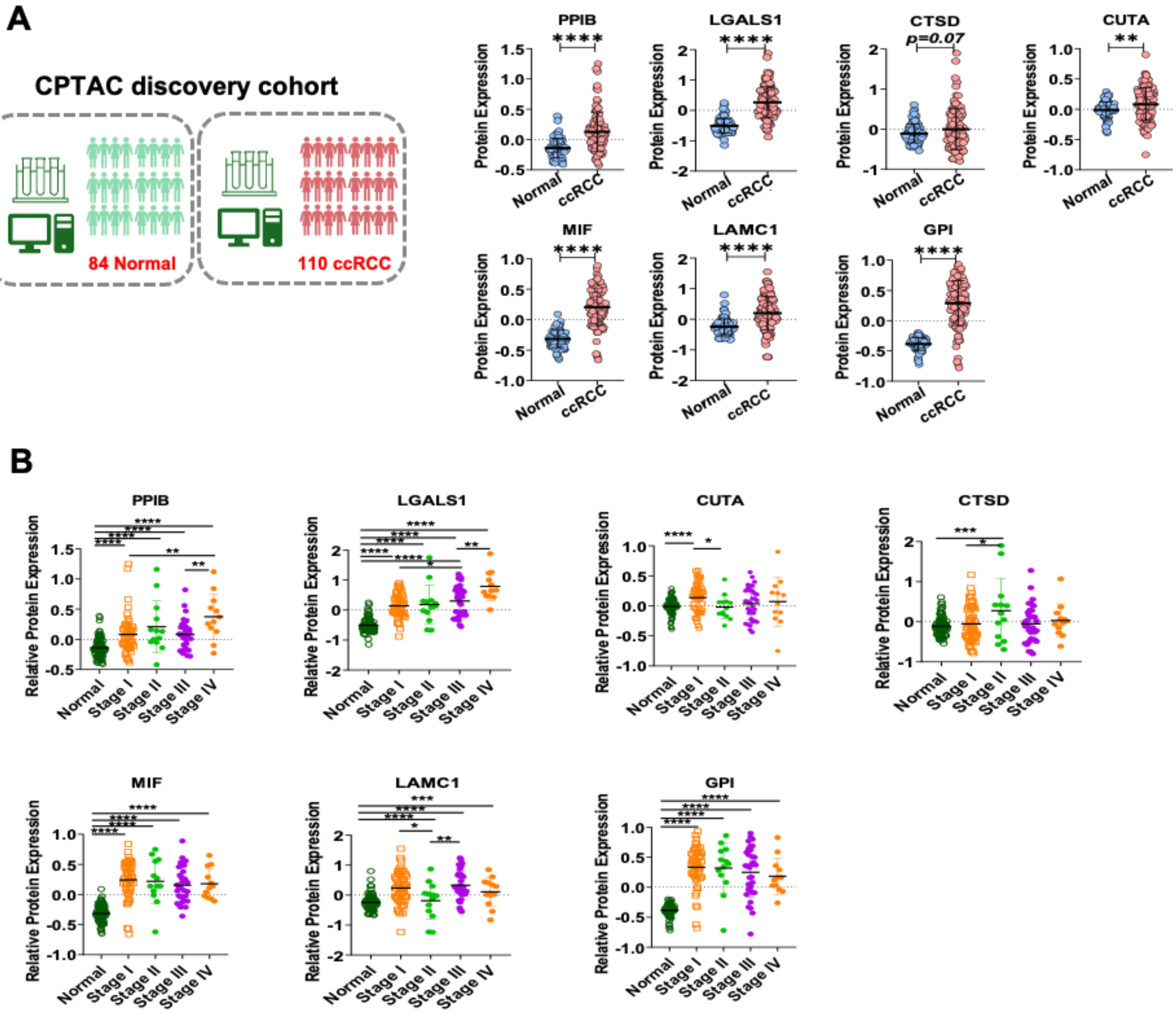

Figure S2 Candidate serum biomarkers identified by proteomics were significantly upregulated in ccRCC tissues compared to normal tissues in the CPTAC ccRCC Discovery and Confirmatory Study (PDC000200 version 2). (A) Protein levels of 7 markers in a cohort of 84 normal and 110 ccRCC patients; (B) Protein levels of 7 markers in this cohort stratified by stage (N=52, 13, 33, 12 for stage 1, 2, 3, and 4, respectively). For (A), a two-tailed Student's t-test was performed. For (B), a one-way ANOVA was performed. *p<0.05; **p<0.01,***p<0.001, ****p<0.0001

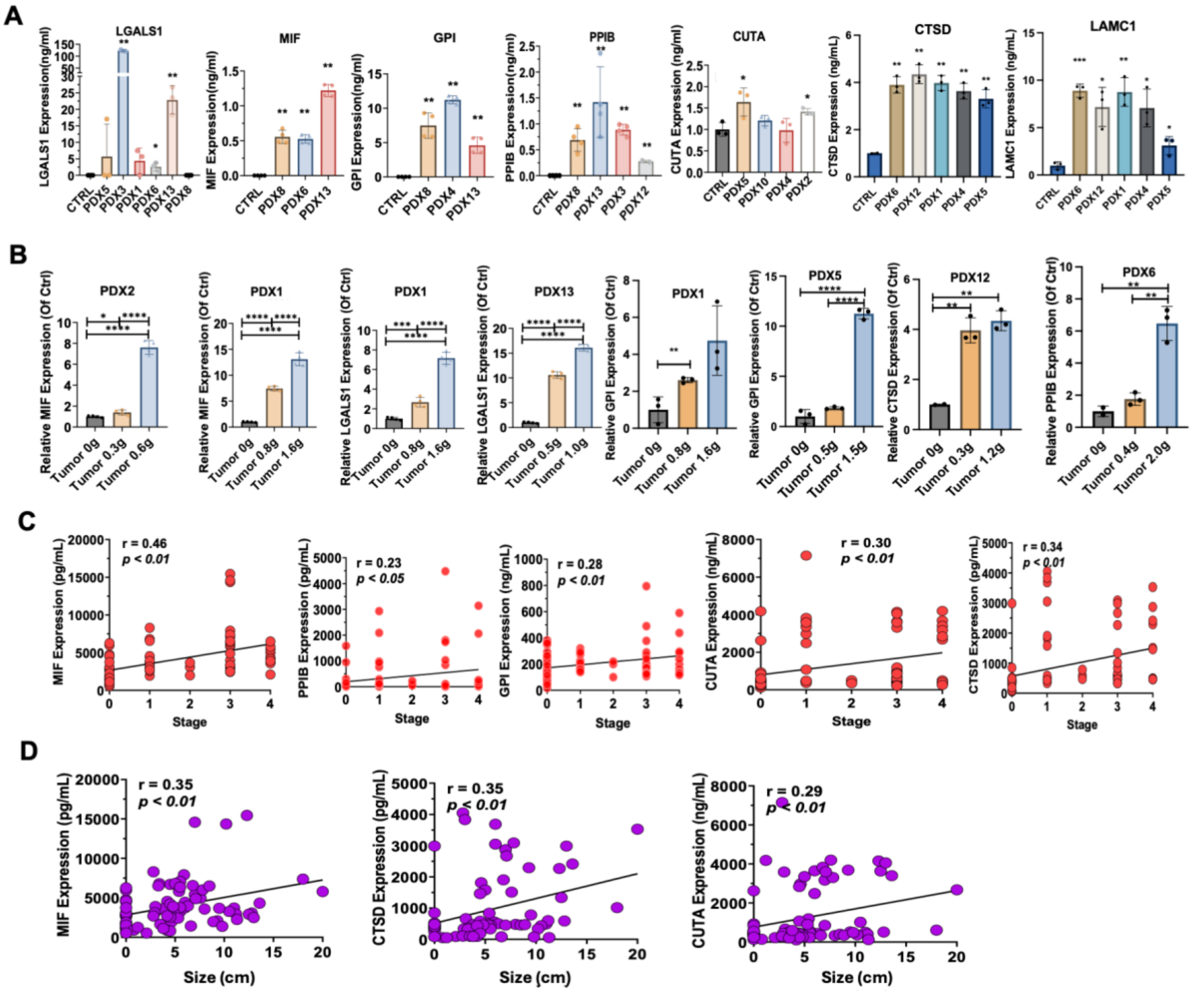

Figure S3 Validation of candidate markers in mouse sera by ELISA and correlation with tumor size and stage in patient cohort. (A) Protein levels of individual markers in mouse sera carrying tumors from different PDX; (B) Protein levels of individual markers in mouse sera carrying tumors with different sizes from the same PDX; (C) Association of serum levels of each candidate marker with size and stage (D) of ccRCC tumors in our cohort. For multiple comparisons in (A) and (B), a one-way ANOVA test followed by Tukey's test was used to correct the multiple comparisons. All error bars represent the mean ± SEM. *p<0.05; **p<0.01,***p<0.001, ****p<0.0001. For (C) and (D), multi-way ANOVA and Pearson correlation analysis were used, respectively.

Table S1 Characteristics of patients included in serum analysis

		**ccRCC**		
**Age**	32-81 yr			
**Sex**	Female N=14	Male N=35		
**Stage**	1 N=17	2 N=3	3 N=19	4 N=10
**Grade**	2 N=18	3 N=14	4 N=16	unknown N=1
**Diameter**	<5cm N=16	5-10 cm N=22	>10 cm N=11	
		**non-ccRCC**		
**Age**	20-74 yr			
**Sex**	Female N=20	Male N=14		
**Condition**	Oncocytoma N=9	Angiomyolipoma N=2	Benign kidney tumor types N=2	Hemorrhagic cyst N=1
	Living kidney donor candidates free of renal neoplasms on imaging N=20			

Methods

Sex as a biological variable.

The PDXs models we used were derived from both male and female patient specimens. We used human donor sex-matched animals to generate PDX tumors. In addition, we validated the candidate biomarkers in a cohort of ccRCC sera from both male and female patients.

Statistics.

Statistical analyses were performed using GraphPad Prism 10.2.3. For multiple comparisons among continuous variables, a one-way ANOVA test followed by Tukey's test was used to correct the multiple comparisons. All error bars represent the mean ± SEM. Notably, p-values are indicated as *, $p < 0.05$; **, $p < 0.01$; ***, $p < 0.001$; ****, $p < 0.0001$; NS, not significant, $p > 0.05$. For correlation analysis, Pearson correlation analysis was used for continuous variables and a multi-way ANOVA was performed for multiple categorical and continuous variables.

Animal study approval.

For animal models, six- to eight-week-old male $RAG2^{-/-}\gamma C^{-/-}$ mice bred in-house at the Stanford University Animal Care Facility were used. The Stanford University Institutional Animal Care and Use Committee at Stanford, California approved all animal studies.

Human tissue and blood samples.

The 32 normal kidney tissues and sera from 49 patients with histologically confirmed ccRCC and 34 patients without ccRCC were obtained by the investigators or by the Stanford University Tissue Bank upon written informed consent from patients under study protocols approved by the

Stanford Institutional Review Board (Stanford IRB #5630) at Stanford, California. Most tissue samples were harvested from primary RCC tumors and adjacent normal kidney that were resected by partial or radical nephrectomy, from resected metastases, or from biopsies of primary or metastatic RCC tumors. In one case, tumor tissue was obtained by the investigators with written consent from a warm autopsy performed after a patient's death from RCC. For PDX generation, fresh tissue samples were cooled on ice until implantation (see below). For proteomic analysis, tissue samples were snap-frozen in liquid nitrogen and stored at -80° C prior to protein extraction. Blood samples were obtained by venipuncture or from vascular access devices, collected in serum tubes, and let coagulate for a minimum time of 30 min. Coagulated blood was then spun at 800 *g* for 10 min of at 360 *g* for 20 min and the liquid supernatant was spun again at 800 g or at 4,122 *g* for 10 min. Cleared serum was then aliquoted, snap-frozen in liquid nitrogen, and stored at -80° C. All samples were processed within 4 hours after collection.

The CPTAC ccRCC Discovery and Confirmatory Study is a research project by the Clinical Proteomic Tumor Analysis Consortium (CPTAC) that focuses on ccRCC. It involves both a discovery phase to identify potential biomarkers and a confirmatory phase to validate them, using proteomic and genomic data[5]. The CPTAC ccRCC dataset provides a landmark integrated proteogenomic characterization of 110 treatment-naive ccRCC and 84 paired-matched normal adjacent tissue (NAT) samples that highlighted a variety of early chromosomal translocation alterations leading to chr3p loss, identified tumor-specific proteomic and phosphoproteomic alterations that are independent of mRNA expression, and defined specific immune-based subtypes based on a combination of mRNA, proteome, and phosphoproteome markers. The

proteomic dataset for this cohort can be downloaded from https://pdc.cancer.gov/pdc/study/PDC000200.

Clinico-demographic data.

Demographic and clinical data was obtained by the investigators under Stanford IRB-approved study protocols by medical record review.

Data availability.

The mass spectrometry proteomics data for 32 normal kidney tissues, ccRCC PDX tissues and sera have been deposited to the ProteomeXchange Consortium via the PRIDE partner repository (https://www.ebi.ac.uk/pride/) with the dataset identifier PXD061075. Other supporting data values and clinical information of patients are available at https://stanfordmedicine.box.com/s/8cdclmdrszng2jjo7e94xa3mh5xag3i0.

Establishing and cryopreserving ccRCC PDX lines

Fresh RCC tissues were obtained from 128 patients undergoing nephrectomy or biopsy between September 2011 and May 2021 at Stanford under an institutional review board–approved protocol with informed consent as described previously[1]. Putative cancers were grossly identified in tumor specimens obtained immediately after surgery. With an automated coring device (Alabama Research and Development, Mundford, AL) under aseptic conditions, 5-mm diameter cores were bored from the putative cancers and submerged in ice-cold HEPES-buffered saline. A Krumdieck tissue slicer (Alabama Research and Development) was used to prepare precision-cut tissue slices according to the manufacturer's instructions. Specifically, each tissue core was

encased in 3% sterile agarose (EMD Chemicals Inc., Hawthorne, NY) inside a mold-plunger assembly specifically devised for tissue embedding before slicing. The embedded tissues were then transferred into the slicer filled with precooled HEPES-buffered saline, and 300-µm slices were cut one at a time. Tissue slices were immediately implanted under the renal capsule of recombination activating gene-2 $(RAG2)^{-/-}\gamma C^{-/-}$ mice (1 slice/mouse) between 6 and 8 weeks of age. Tumor growth was monitored by weekly MRI. tumors were harvested once their volumes reached 1 cm^3, and precision-cut as described above. Tumor tissue slices were cryopreserved in 95% FBS+5% DMSO and stored in a liquid nitrogen tank for future experiments.

Generation of ccRCC PDX xenographs.

All procedures involving animals and their care were approved by the Institutional Animal Care and Use Committee of Stanford University in accordance with institutional and National Institutes of Health guidelines. Ten of the eleven PDX lines that were used in this study were established and passaged at Stanford University from ccRCC tissues obtained from patients undergoing nephrectomy, autopsy, or biopsy between September 2011 and February 2021 at Stanford University under an institutional review board–approved protocol with informed consent as previously described[1]. One PDX line was obtained from the NCI Patient-Derived Models Repository (PDMR), patient ID 597326. For each PDX, frozen slices of PDX tumor tissue were thawed (from passages 3-6) and implanted under the renal capsule of each of fifteen $RAG2^{-/-}\gamma C^{-/-}$ mice per PDX line. Male or female mice were used according to the sex of the tissue donor. Tumor growth was monitored by conventional proton MRI using a Discovery MR901 7.0-Tesla MRI system (Agilent Technologies, Santa Clara, CA) at the Stanford University Small Animal Imaging Facility per our published protocol[1]. Three-dimensional

volumetric modeling was performed with OsiriX 4.1 (Pixmeo, Bernex, Switzerland) to calculate xenograft tumor volumes. Groups of 5 mice were sacrificed when tumor volumes reached 0.5, 1.0, and 1.5 cm^3 by MRI. Tumor tissues were harvested and weighed. Sera from all tumor-bearing mice and five non-engrafted isogenic mice as negative controls were collected, snap-frozen in liquid nitrogen, and stored at -80° C.

Proteomic profiling by liquid chromatography-mass spectrometry (LC-MS).

Sera from mice with a tumor volume of 1.5 cm^3 were used for proteomic profiling. For each PDX line and negative control, 3 out of the 5 sera with the lowest contamination with hemoglobin due to red blood cell lysis during sample collection by visualization were chosen for subsequent analysis. Protein extraction from tumor tissues and 32 normal human kidney tissues was performed as described previously [2]. Serum samples were immunodepleted to remove three abundant proteins using a Multiple Affinity Removal Column Mouse 3 (Agilent, Santa Clara, CA) according to the manufacturer's instructions. Protein concentrations were determined by BCA protein assay according to manufacturer instruction (Thermo Fisher Scientific, Waltham, MA, USA). Abundant plasma proteins were depleted from sera using an immuno-depletion column containing antibodies against these abundant proteins as described previously to reduce the sample complexity[2]. 25 µg of protein were reduced, alkylated, and fragmented with trypsin (Thermo Fisher Scientific) as described previously[2]. The resulting tryptic peptides were reconstituted in 0.1% formic acid (50 µl, Fisher Scientific), fractionated using a Dionex Ultimate Rapid Separation liquid chromatography system (Thermo Fisher Scientific), and analyzed on an Orbitrap Tribrid Eclipse mass spectrometer, equipped with an Orbitrap mass analyzer as described previously[2].

Proteomic data analysis

Raw data was processed through a two-stage search using Byonic 4.0.12. The initial stage involved a search against the Swiss-Prot database consisting of the reference human proteome (as of 2022; 20,645 entries). This was followed by a second-stage search, which utilized the Swiss-Prot database containing the mouse reference proteome (as of 2022; 17,380 entries). The search parameters included trypsin digestion, allowing for a maximum of two missed cleavages, a precursor mass tolerance of 0.5 Da, and a fragment mass tolerance of 10 ppm. The parameters also specified fixed cysteine carbamidomethylation and variable modifications including methionine oxidation and asparagine deamination. To maintain the integrity of the peptide identification process, peptides with a false discovery rate (FDR) greater than 1% were excluded from the results. Any peptides identified in the human and mouse database searches were discarded to focus on non-homologous peptides. (Human kidney tissue data was only searched against the human database and no subsequent homology analysis was performed.) An analysis of the human-identified proteins versus homologous mouse peptides was performed using a custom R script. Protein concentrations were determined by evaluating the unique signals associated with each protein within the samples. The relative protein abundance was ascertained by comparing these specific signals to the mean signal observed across all samples, thus providing an estimate of each protein's relative abundance within the complete protein content of the experimental dataset. A normalization procedure was carried out to ensure comparability across samples and enable statistical analysis. This process adjusted the relative abundance values to a normal distribution with a mean of 0 and a standard deviation of 1. The final statistical analysis was conducted using

the Student's t-test. Only proteins that exhibited a p value smaller than 0.01 were included in the subsequent analysis stages.

We identified 785 proteins that were significantly higher in ccRCC PDX tissue compared to 32 normal human kidney tissues. These proteins constitute 22.5% of the total proteins detected in our tissue proteomic analysis. In other words, a total of 3,488 proteins were measured, and out of these, 785 were upregulated in ccRCC PDX tissues. These 3,488 proteins and raw proteomics data were deposited to the ProteomeXchange Consortium via the PRIDE partner repository (https://www.ebi.ac.uk/pride/) with the dataset identifier PXD061075. The number of proteins detected was comparable to previous mass spectrometry-based proteomic studies using fresh ccRCC tissues[3,4].

Secreted human proteins were identified using the Secretome database (https://www.proteinatlas.org/humanproteome/tissue/secretome). The Secretome database annotated 1,903 human secreted proteins. Our proteomic analysis identified 3,488 proteins in ccRCC tissues, out of which 374 were found in the Secretome database, which is comparable to the number of human serum proteins identified using mass spectrometry in previous studies[5,6].

For 110 ccRCC tissues and 84 adjacent normal tissues in the CPTAC ccRCC Discovery and Confirmatory Study (PDC000200 version 2), proteomic data were downloaded from Proteomic Data Commons (https://pdc.cancer.gov/pdc/study/PDC000200).

Determination of serum protein levels by ELISA.

ELISA kits for candidate biomarkers were purchased from the following sources:

LEGEND MAX Human Active MIF ELISA Kit (Cat # 438407) from Biolegend (San Diego, CA, USA); Human Galectin-1/LGALS1 ELISA Kit (Cat # EH203RB) from ThermoFisher (Waltham, MA); Human Cyclophilin B/SCYLP ELISA (Cat # ELH-CYPB-1) from RayBiotech (Norcross, GA, USA); Human GPI (Glucose-6-Phosphate Isomerase) ELISA Kit (Cat # XPEH3165) from Xpressbio (Frederick, MD, USA); Human Laminin subunit gamma-1 (LAMC1) Elisa Kit (Cat # EK712845); Human Cathepsin D (cath-D) Elisa Kit (Cat # EK712042) and Human Protein CutA (CUTA) Elisa Kit (Cat # EK712044) from AFG Bioscience (Northbrook, IL, USA). For both human and mouse samples, 25 μL sera was diluted 1:4 according to the manufacturer's instructions. Triplicate samples were used for each serum. Concentrations of proteins of interest in serum were determined using a standard curve according to the manufacturer's instructions.

Model construction and validation.

Stratified random sampling was performed to separate the 34 non-ccRCC donors and 49 ccRCC patients into the training (32 ccRCC and 22 non-ccRCC donors) and test dataset (17 ccRCC and 12 non-ccRCC donors). A sparse linear regression model was generated using "cv.glmnet" in R with 5-fold cross-validation. AUC was calculated using "roc" in R and a risk score computed using levels of six candidate markers (4.424e-04 x MIF+2.259e-04 x LGALS1 + 4.19e-04 x PPIB + 5.020e-05 x CTSD + 2.7e-05 x CUTA + 5.162e-03 x GPI).

Limitations of the study

There are several limitations in our study. First, the sensitivity of LC-MS-based proteomics analysis is suboptimal[7]. For example, not all proteins are digested with equal efficiency, which can

bias protein identification and quantification. In addition, while LC-MS/MS has a good dynamic range, it may not be sufficient for detecting very low-abundance proteins or highly variable proteins. Second, the sample sizes of ccRCC tissues and sera we used in this study are small, which may lead to reduced statistical power, increased variability, and difficulty in detecting small effects[8]. We are in the process of validating the candidate markers in additional serum samples from ccRCC patients. Third, the candidate selection criteria of 3 out of 8 PDXs (37.5%) are arbitrary, which may lead to potential biases by excluding biomarkers expressed in a smaller percentage of patients.

Additional discussion

Additional studies are needed to uncover the mechanisms of action of the identified markers in ccRCC, which will be the focus of our future investigations. These markers may play critical roles in ccRCC development and progression. Several of them have been implicated in ccRCC cellular functions and patient prognosis, albeit the results were contradictory in some cases. For instance, knocking down MIF inhibited cell migration in cultured ccRCC cells[9]. In addition, ccRCC patients in the TCGA cohort with higher MIF expression had significantly shorter overall survival[10], while negative MIF expression was associated with poor outcomes in a cohort of 152 Korean ccRCC patients[9]. LAMC1 expression was positively correlated with infiltration of CD4(+) T cells, macrophages, and neutrophils, as well as survival in ccRCC patients[11]. Higher LGALS1 expression predicted worse survival in multiple cohorts of ccRCC patients[12,13], however, higher LGALS1 was associated with better response to anti-PD-1 treatment[14]. Both GPI and CTSD served as poor prognosis markers in ccRCC, suggesting they may play a promoting role in ccRCC

progression[15,16]. The role of CUTA and PPIB in ccRCC has not been reported. We hope our future investigations will help reveal the functions of these biomarkers in ccRCC biology.

Acknowledgments

This work is supported by the NIH NCI grants 1R21CA256271 and 5R21CA276896, and W81XWH2210651 from the Department of Defense. This work is subject to the NIH Public Access Policy. Through acceptance of this federal funding, the NIH has been given a right to make the work publicly available in PubMed Central.

Author contributions

The order of the co-first authors, DZ and CLC was determined by a coin toss.

Other accompanying files

Supporting data values and clinical information of patients are available at https://stanfordmedicine.box.com/s/8cdclmdrszng2jjo7e94xa3mh5xag3i0.

References

1. Thong, A.E. *et al.* Tissue slice grafts of human renal cell carcinoma: an authentic preclinical model with high engraftment rate and metastatic potential. *Urol Oncol* **32**, 43.e23-30 (2014).
2. Wen, R.M. *et al.* Sialylated glycoproteins suppress immune cell killing by binding to Siglec-7 and Siglec-9 in prostate cancer. *J Clin Invest* (2024).

3. Park, J. *et al.* Using Comparative Proteomics to Identify Protein Signatures in Clear Cell Renal Cell Carcinoma. *Cancer Genomics Proteomics* **20**, 592-601 (2023).

4. Sun, X. *et al.* Comparative proteomic profiling identifies potential prognostic factors for human clear cell renal cell carcinoma. *Oncol Rep* **36**, 3131-3138 (2016).

5. Adkins, J.N. *et al.* Toward a human blood serum proteome: analysis by multidimensional separation coupled with mass spectrometry. *Mol Cell Proteomics* **1**, 947-55 (2002).

6. Pieper, R. *et al.* The human serum proteome: display of nearly 3700 chromatographically separated protein spots on two-dimensional electrophoresis gels and identification of 325 distinct proteins. *Proteomics* **3**, 1345-64 (2003).

7. Birhanu, A.G. Mass spectrometry-based proteomics as an emerging tool in clinical laboratories. *Clinical Proteomics* **20**, 32 (2023).

8. Hamad, A.A. & Ahmed, S.K. Understanding the Lower and Upper Limits of Sample Sizes in Clinical Research. *Cureus* **17**, e76724 (2025).

9. An, H.J., Koh, H.M., Lee, J.S. & Song, D.H. Prognostic role of macrophage migration inhibitory factor in patients with clear cell renal cell carcinoma. *Medicine (Baltimore)* **99**, e23277 (2020).

10. Parol-Kulczyk, M. *et al.* Macrophage migration inhibitory factor (MIF) predicts survival in patients with clear cell renal cell carcinoma. *J Pathol Clin Res* **10**, e12365 (2024).

11. Bai, J. *et al.* Comprehensive analysis of LAMC1 expression and prognostic value in kidney renal papillary cell carcinoma and clear cell carcinoma. *Front Mol Biosci* **9**, 988777 (2022).

12. Fang, J. *et al.* LGALS1 was related to the prognosis of clear cell renal cell carcinoma identified by weighted correlation gene network analysis combined with differential gene expression analysis. *Front Genet* **13**, 1046164 (2022).

13. Seubwai, W., Sangkhamanon, S. & Zhang, X. Identification of IGFBP3 and LGALS1 as potential secreted biomarkers for clear cell renal cell carcinoma based on bioinformatics analysis and machine learning. *Adv Clin Exp Med* (2025).

14. Li, Y. *et al.* Unraveling LGALS1 as a Potential Immune Checkpoint and a Predictor of the Response to Anti-PD1 Therapy in Clear Cell Renal Carcinoma. *Pathol Oncol Res* **26**, 1451-1458 (2020).

15. Lucarelli, G. *et al.* Increased Expression of the Autocrine Motility Factor is Associated With Poor Prognosis in Patients With Clear Cell-Renal Cell Carcinoma. *Medicine (Baltimore)* **94**, e2117 (2015).

16. Mourão, T.C. *et al.* Prognostic role of the immunohistochemical expression of proteins related to the renin-angiotensin system pathway in nonmetastatic clear cell renal cell carcinoma. *Urol Oncol* **41**, 359.e1-359.e13 (2023).
